# Oral anticoagulant persistence in patients with non-valvular atrial fibrillation: A cohort study using primary care data in Germany

**DOI:** 10.1371/journal.pone.0185642

**Published:** 2017-10-10

**Authors:** Shuk-Li Collings, Cinira Lefèvre, Michelle E. Johnson, David Evans, Guido Hack, Gillian Stynes, Andrew Maguire

**Affiliations:** 1 OXON Epidemiology, London, United Kingdom; 2 Centre for Observational Research and Data Sciences, Bristol-Myers Squibb, Paris, France; 3 Medical Department, Bristol-Myers Squibb, Munich, Germany; 4 Worldwide Health Economics & Outcomes Research, Bristol-Myers Squibb, London, United Kingdom; University of Bologna, ITALY

## Abstract

This study examined characteristics and treatment persistence among patients prescribed oral anticoagulants (OACs) for stroke prevention in non-valvular atrial fibrillation (NVAF). We identified 15,244 patients (51.8% male, 72.7% aged ≥70) with NVAF and no prior OAC therapy who were prescribed apixaban (n = 1,303), rivaroxaban (n = 5,742), dabigatran (n = 1,622) or vitamin-K antagonists (VKAs, n = 6,577) between 1-Dec-2012 and 31-Oct-2014 in German primary care (IMS® Disease Analyzer). We compared OAC persistence using Cox regression over patients’ entire follow-up and using a data-driven time-partitioned approach (before/after 100 days) to handle non-proportional hazards. History of stroke risk factors (stroke/transient ischaemic attack [TIA] 15.2%; thromboembolism 14.1%; hypertension 84.3%) and high bleeding risk (HAS-BLED score≥3 68.4%) was common. Apixaban-prescribed patients had more frequent history of stroke/TIA (19.7%) and high bleeding risk (72.6%) than other OACs. 12-month persistence rates were: VKA 57.5% (95% confidence interval (CI) 56.0–59.0%), rivaroxaban 56.6% (54.9–58.2%), dabigatran 50.1% (47.2–53.1%), apixaban 62.9% (58.8–67.0%). Over entire follow-up, compared to VKA, non-persistence was similar with apixaban (adjusted hazard ratio 1.08, 95% CI 0.95–1.24) but higher with rivaroxaban (1.21, 1.14–1.29) and dabigatran (1.53, 1.40–1.68). Using post-hoc time-partitioned approach: in first 100 days, non-persistence was higher with apixaban (1.37, 1.17–1.59), rivaroxaban (1.41, 1.30–1.53) and dabigatran (1.91, 1.70–2.14) compared to VKA. Compared to apixaban, rivaroxaban non-persistence was similar (1.03, 0.89–1.20), dabigatran was higher (1.39, 1.17–1.66). After 100 days, apixaban non-persistence was lower than VKA (0.66, 0.52–0.85); rivaroxaban (0.97, 0.87–1.07) and dabigatran (1.10, 0.95–1.28) were similar to VKA. Furthermore, rivaroxaban (1.46, 1.13–1.88) and dabigatran (1.67, 1.26–2.19) non-persistence was higher than apixaban. This study describes real-world observations on OAC use, particularly early apixaban use following approval for NVAF, in Germany. We identified potential differential OAC prescribing and higher persistence with apixaban than other OACs after 100 days’ treatment. Larger studies are needed with longer follow-up to establish long-term patterns.

## Introduction

Atrial fibrillation (AF) is the most common sustained cardiac arrhythmia [1], with an estimated global burden of over 30 million people [2] and an increasing prevalence over time [3]. While anti-arrhythmic drugs can be prescribed to treat the irregular heart beat patterns that characterise AF, it is a condition which carries an increased likelihood of different sequelae [4]. In particular, there is an elevated risk of ischaemic stroke in patients with AF [5], for which long-term treatment with oral anticoagulants (OACs) is recommended to prevent stroke [6–9].

Traditionally, vitamin K antagonists (VKAs) have been the preferred OAC; however, VKAs have a narrow therapeutic window, require close monitoring and come with substantial dietary restrictions [10]. These limitations may explain the poor persistence rates documented with over a quarter of users stopping VKAs within a year of initiation [11,12]. In recent years, the novel OACs (NOACs) dabigatran, rivaroxaban, apixaban and edoxaban have become available. While clinical trials have shown the NOACs to have at least equal efficacy to VKA [6–8,13], the NOACs are designed to be simpler to use in that they do not require the patient to be monitored or control their diet [14,15]. Moreover, there is evidence that subsequent bleeding events–one of the major concerns of OAC use–are less frequent with apixaban, adjusted dose dabigatran and edoxaban than with VKAs in clinical trials [8,13,16].

As patients with AF are at elevated risk of ischaemic stroke when untreated, assessing OAC persistence is crucial to understanding the extent to which patients continuously receive the benefit of stroke prevention while tolerating possible side effects. Research using real-world data is key to observing persistence without the influence of a study environment, such as a clinical trial. However, real-world data studies of new drugs require time for information to naturally accumulate as the drugs become routinely used in clinical practice. At the time this study was performed in 2015, apixaban was the most recently approved OAC licensed for stroke prevention in NVAF. With rivaroxaban and dabigatran having also been in circulation for some years, it is now timely to use real-world data to assess OAC treatment persistence.

The aim of this study was therefore to describe the characteristics of patients newly prescribed different OACs for stroke prevention and then to estimate and compare persistence rates between OACs using real-world data from German primary care.

## Methods

This was a cohort study of patients with NVAF who were prescribed an OAC using primary care data in Germany and who were naïve to OAC therapy i.e. had no prior records of OAC therapy.

### Data source

The study used German primary care data from IMS® Disease Analyzer. The German IMS® Disease Analyzer data contain anonymised medical records of patient visits to a primary care physician for approximately 7% of the German population. It contains information recorded during routine clinical practice, e.g. medical diagnoses, prescriptions issued and diagnostic tests. Information from IMS® Disease Analyzer were validated in a study by Becher *et al* who found prescription and diagnosis data to be concordant with national statistics, and that the distribution of age and region of participating physicians were similar to figures obtained from the German Medical Association [17]. However, the data are accumulated from the information recorded at each patient visit to the same primary care physician. In Germany, patients have the freedom to move between health care professionals, thus consultations with a different primary care physician will not be identifiable and, moreover, consultations with other health care professionals outside of primary care, including AF specialists such as cardiologists, are not included in these data. This study is registered as an observational study on ClinicalTrials.gov (identifier NCT02488421).

### Study population

We identified adults with NVAF who were prescribed an OAC during the study period (1st December 2012 to 31st October 2014) and who were OAC-naïve i.e. patients had no records of OAC therapy in their data prior to the index date. Index date was the date of the first prescription of each OAC in the study period. We included patients with a record of AF and without a record for a valvular condition (moderate-severe mitral stenosis and prosthetic heart valves) on or before index date. All patients were aged 18 years or older at the index date and had at least 12 months since their first observed visit in the data (i.e. at least 12 months between first ever visit to their primary care physician and index date). We also planned and analysed the wider population of all patients initiating OACs in the study period which includes those who had been previously prescribed other OACs and provide these results as supplementary information (S1 and S2 Tables).

### Patient characteristics

In each OAC cohort, we described patients’ age, gender, region (physician’s practice), time since AF diagnosis, history of stroke risk factors, baseline stroke risk measured by the CHA_2_DS_2_VASc score [≥ 2 indicates high stroke risk], history of bleeding events, baseline bleeding risk measured by the HAS-BLED score [≥ 3 indicates high bleeding risk] and concomitant medication use (prescribed on index date or within 3 months after index date). We also described the dose of each NOAC at index date, classifying prescriptions of 5 mg apixaban, 20 mg rivaroxaban and 150 mg dabigatran as ‘standard’ doses and 2.5 mg apixaban, 15 mg rivaroxaban, and 110mg dabigatran as ‘adjusted’ doses, in line with the recommendations from the corresponding Summary of Product Characteristics (SmPC) [18–20]. A small proportion of patients with NVAF (less than 3%) were prescribed 10 mg rivaroxaban or 75 mg dabigatran on index date. While these are not licensed doses for NVAF, it was assumed they were prescribed for this indication and, since the dose is lower than the standard recommended for NVAF, these doses were grouped with the ‘adjusted’ doses.

### Definition of OAC treatment persistence

We grouped consecutive prescriptions of the same OAC for each patient from the first record in their history to the end of follow-up (i.e. earliest of end of study period or last observation date) to form OAC-specific treatment lines. We estimated the duration of each prescription assigning prescription end dates based on the recorded quantity prescribed and applying a daily dose of twice a day for apixaban and dabigatran, once a day for rivaroxaban and VKA respectively. We calculated the gap between the end of each prescription and the date of the next prescription and defined a particular OAC treatment line as discontinued if:

- the gap was greater than 60 days, and
- there were more than 60 days between the end of the treatment line and the end of follow-up.

A 60 day gap was considered a clinically relevant length of time, as per Zalesak *et al*, in which a patient is expected to obtain their next prescription if they choose to continue treatment [11]. A switch was defined if a different OAC was prescribed during the course of the index OAC regimen or in the 60 days following the end of the index treatment line, thus follow-up was censored for the initial OAC at the date of the first prescription of the next OAC.

VKA treatment lines were extended if there was a record of an INR test after the index date in order to account for possible additional prescriptions or changes to dosage made outside of primary care during VKA patients’ routine monitoring of INR. Where an INR test was recorded in the data, it was assumed that the patient received a VKA prescription of 100 days (median length of observed VKA prescription in the data). This method ensures a generous estimation of VKA persistence compared to the use of prescription records only.

For each OAC treatment line, we defined patients as persistent on their OAC treatment if there was no regimen change (i.e. discontinuation or switch) during follow-up. Persistence was not assessed for treatment lines with an insufficient amount of follow-up data (equivalent to or less than the length of the specified discontinuation gap, i.e. 60 days or less, between index date and end of follow-up).

### Statistical analyses

For the patient characteristics, categorical data are summarised by the number and percentage of patients in each category and continuous data are summarised by the number of patients, mean and standard deviation or median with lower and upper quartiles.

Cumulative incidence curves described time to non-persistence between OAC cohorts. Persistence rates were calculated as 100% minus the cumulative incidence of non-persistence for each OAC cohort over the entire follow-up period and at specific time points (3, 6 and 12 months) of follow-up, along with the number of patients at risk and the number censored. While death presents a competing risk for non-persistence, this was not accounted for in the analyses due to low recording of deaths in the data.

We used Cox regression models to compare time to non-persistence while adjusting for differences in patients’ baseline characteristics. We examined Schoenfeld residuals to assess the proportional hazards assumption and, upon finding evidence of non-proportionality, performed a post-hoc time-partitioned analysis. In this, we partitioned follow-up at 100 days based on inspection of the cumulative incidence curves. Based on the Schoenfeld residuals, the proportionality assumption was satisfied before and after 100 days. We reported HRs and 95% confidence intervals (CIs) on the non-partitioned analysis, where they represent the average HR over all follow-up time, and for the partitioned analyses separately.

We adjusted HRs for baseline characteristics and variables of clinical interest using significance-based backward selection approach, retaining only variables with evidence at the 5% level of being associated with persistence. The full, starting, model from which the adjusted model was derived comprised of gender, age at index date, region (East or West Germany), history of stroke risk factors (stroke or transient ischaemic attack [TIA], thromboembolism, congestive heart failure, vascular disease, hypertension, diabetes), history of any bleeding, history of liver disease, and concomitant therapies. All patients had a record of age, gender and region. Patients who did not have a record for the remaining comorbidities and therapies of interest were assumed to not have the condition or therapy.

All analyses were conducted in SAS version 9.4 (SAS Institute Inc., Cary, NC, USA).

### Sensitivity analyses

We performed sensitivity analyses to examine the impact of the assumptions used in our persistence analyses. We recalculated persistence rates at specific time points in each of the following scenarios: 1) for patients who regularly visited the same physician in the 12 months prior to index date (i.e. visited at least once every 3 months) to identify whether non-persistence may have been associated with patients’ infrequent visits to the same physician, 2) non-persistence using a 30 day discontinuation gap instead of a 60 day gap, and 3) VKA treatment lines without the inclusion of INR records to extend VKA treatment lines.

## Results

### Cohort allocation

There were 15,244 adults with NVAF who were newly prescribed an OAC during the study period. VKA was newly prescribed to 43.1% (n = 6,577) of patients; rivaroxaban to 37.7% (n = 5,742), dabigatran to 10.6% (n = 1,622) and apixaban to 8.5% (n = 1,303) (Table 1). Of the NOAC cohorts, an adjusted dose was prescribed on index date to 35.7% of the apixaban cohort, 35.0% of the rivaroxaban cohort, and 53.4% of the dabigatran cohort. Over the study period, new use of VKA and dabigatran declined, rivaroxaban use was stable and apixaban increased.

**Table 1:** Patient characteristics.

	All study population	Apixaban	Rivaroxaban	Dabigatran	VKA
	N = 15,244	N = 1,303	N = 5,742	N = 1,622	N = 6,577
**Gender (n, %)**					
Male	7,895 (51.8%)	673 (51.7%)	2,858 (49.8%)	841 (51.8%)	3,523 (53.6%)
Female	7,349 (48.2%)	630 (48.3%)	2,884 (50.2%)	781 (48.2%)	3,054 (46.4%)
**Region (n, %)**					
West Germany	12,397 (81.3%)	1,081 (83.0%)	4,540 (79.1%)	1,289 (79.5%)	5,487 (83.4%)
East Germany	2,847 (18.7%)	222 (17.0%)	1,202 (20.9%)	333 (20.5%)	1,090 (16.6%)
**Age (years) at index date**					
≥ 70 years (n, %)	11,076 (72.7%)	985 (75.6%)	4,066 (70.8%)	1,125 (69.4%)	4,900 (74.5%)
Median (IQR)	75 (69–81)	76 (70–83)	75 (68–81)	75 (67–81)	75 (69–81)
**Time (months) between AF diagnosis and index date**					
Median (IQR)	0.5 (0.0–16.1)	0.4 (0.0–17.3)	0.5 (0.0–18.8)	0.4 (0.0–13.6)	0.5 (0.0–15.0)
**Index NOAC dose (n, %)**	N = 8,667				
Adjusted dose^ᴪ^	3,339 (38.5%)	465 (35.7%)	2,008 (35.0%)	866 (53.4%)	-
**History of stroke risk factors (n, %)**					
Stroke or transient ischaemic attack	2,310 (15.2%)	257 (19.7%)	844 (14.7%)	272 (16.8%)	937 (14.2%)
Thromboembolism	2,143 (14.1%)	138 (10.6%)	863 (15.0%)	182 (11.2%)	960 (14.6%)
Congestive heart failure	5,249 (34.4%)	460 (35.3%)	1,935 (33.7%)	509 (31.4%)	2,345 (35.7%)
Vascular disease	8,338 (54.7%)	716 (55.0%)	3,048 (53.1%)	839 (51.7%)	3,735 (56.8%)
Hypertension	12,846 (84.3%)	1,113 (85.4%)	4,803 (83.6%)	1,370 (84.5%)	5,560 (84.5%)
Diabetes	5,689 (37.3%)	481 (36.9%)	2,084 (36.3%)	589 (36.3%)	2,535 (38.5%)
**CHA**_**2**_**DS**_**2**_**-VASc score at index date (n, %)**					
< 2	937 (6.1%)	73 (5.6%)	435 (7.6%)	123 (7.6%)	306 (4.7%)
≥ 2	14,307 (93.9%)	1,230 (94.4%)	5,307 (92.4%)	1,499 (92.4%)	6,271 (95.3%)
**History of events (n, %)**					
Gastrointestinal ulceration	952 (6.2%)	68 (5.2%)	363 (6.3%)	99 (6.1%)	422 (6.4%)
Gastrointestinal bleeding	2,363 (15.5%)	202 (15.5%)	925 (16.1%)	270 (16.6%)	966 (14.7%)
Other bleeding^¶^	1,167 (7.7%)	94 (7.2%)	453 (7.9%)	123 (7.6%)	497 (7.6%)
Any bleeding^¶^	3,412 (22.4%)	289 (22.2%)	1,325 (23.1%)	373 (23.0%)	1,425 (21.7%)
**HAS-BLED score**^**#**^ **at index date (n, %)**					
< 3	4,814 (31.6%)	357 (27.4%)	1,745 (30.4%)	547 (33.7%)	2,165 (32.9%)
≥ 3	10,430 (68.4%)	946 (72.6%)	3,997 (69.6%)	1,075 (66.3%)	4,412 (67.1%)
**Concomitant therapy**^**^**^ **(n, %)**					
Parenteral anticoagulants	1,272 (8.3%)	29 (2.2%)	166 (2.9%)	58 (3.6%)	1,019 (15.5%)
Antiplatelet	1,574 (10.3%)	101 (7.8%)	538 (9.4%)	151 (9.3%)	784 (11.9%)
Aspirin monotherapy	1,253 (8.2%)	79 (6.1%)	461 (8.0%)	118 (7.3%)	595 (9.0%)
Other antiplatelet therapies^ⱡ^	630 (4.1%)	34 (2.6%)	167 (2.9%)	54 (3.3%)	375 (5.7%)
Anti-arrhythmic	1,878 (12.3%)	161 (12.4%)	773 (13.5%)	195 (12.0%)	749 (11.4%)
Beta-blocker	9,700 (63.6%)	775 (59.5%)	3,662 (63.8%)	1,022 (63.0%)	4,241 (64.5%)
Statin	3,985 (26.1%)	353 (27.1%)	1,380 (24.0%)	422 (26.0%)	1,830 (27.8%)
Antidiabetic agent	2,369 (15.5%)	183 (14.0%)	833 (14.5%)	236 (14.5%)	1,117 (17.0%)
Antihypertensive agent	10,222 (67.1%)	875 (67.2%)	3,688 (64.2%)	1,079 (66.5%)	4,580 (69.6%)
Proton pump inhibitor	4,544 (29.8%)	357 (27.4%)	1,710 (29.8%)	509 (31.4%)	1,968 (29.9%)

ᴪ Adjusted dose defined as follows: apixaban 2.5mg, rivaroxaban 10/15mg or dabigatran 75/110mg prescribed on index date.

¶ Other bleeding includes intraocular, pericardial, urinary, intra-articular and lung bleedings. Any bleeding includes gastrointestinal, intracranial and other bleeding.

# Labile international normalised ratio is also a component of the HAS-BLED score but was not included as there is incomplete recording in IMS® Disease Analyzer. The HAS-BLED score therefore ranges from 0 to 8. High alcohol intake has been included in the HAS-BLED score however is likely under-recorded in IMS® Disease Analyzer.

^ Concomitant therapy: prescribed on index date or within 3 months after index date.

ⱡ Other antiplatelet therapy includes abciximab, clopidogrel, dipyridamole, prasugrel, ticagrelor, ticlopidine and tirofiban.

### Patient characteristics

Just over half of the study population were male (51.8%) and nearly three-quarters were 70 years or older (72.7%, Table 1).

**Fig 1:**
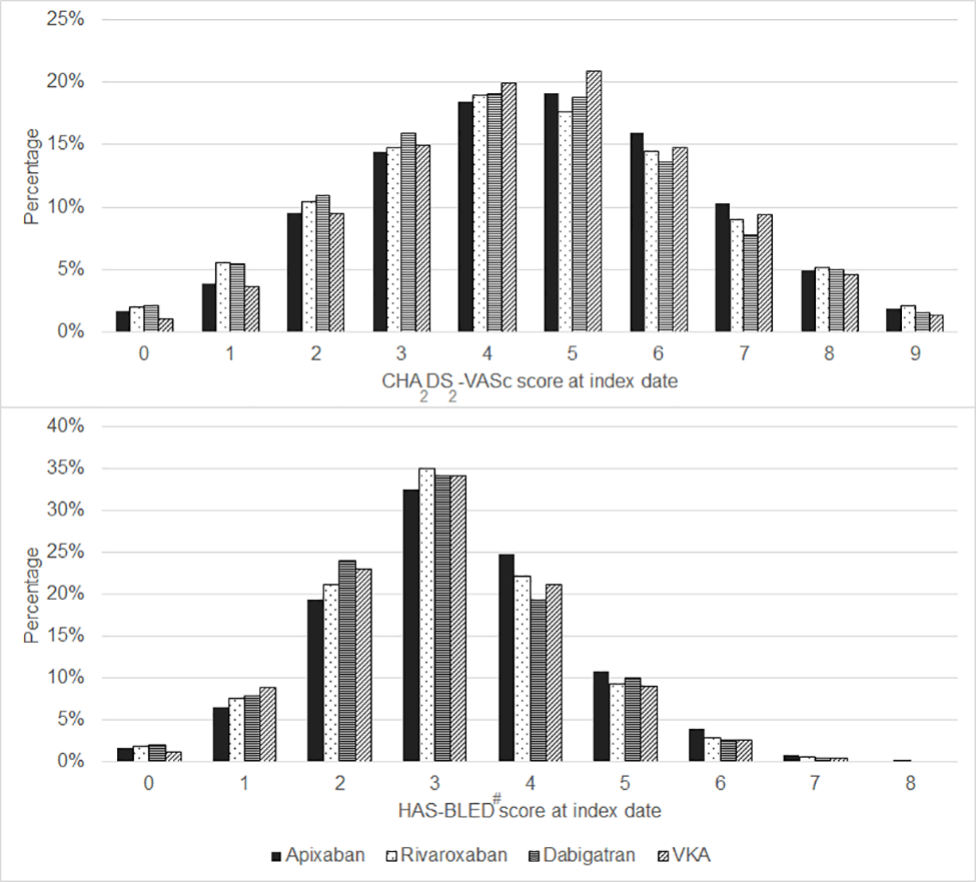
Distribution of CHA_2_DS_2_-VASc and HAS-BLED scores. # Labile international normalised ratio is also a component of the HAS-BLED score but was not included as there is incomplete recording in IMS® Disease Analyzer. The HAS-BLED score therefore ranges from 0 to 8. High alcohol intake has been included in the HAS-BLED score however is likely under-recorded in IMS® Disease Analyzer.

**Fig 2:**
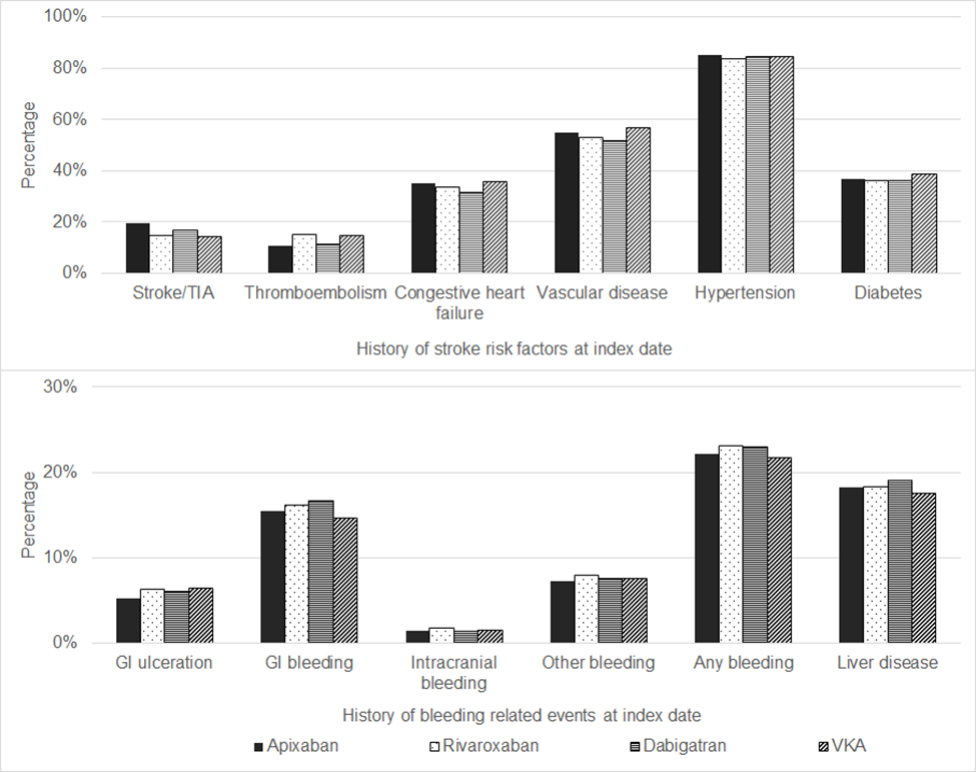
History of stroke risk factors and bleeding events. TIA = transient ischaemic attack; GI = gastrointestinal.

Overall, 93.9% (n = 14,307) had a high stroke risk (i.e. CHA_2_DS_2_VASc score ≥ 2) and stroke risk was similar across OAC cohorts (Table 1 and Fig 1). Stroke risk factors were common; overall, 84.3% had a history of hypertension, 54.7% vascular disease, 37.3% diabetes, 34.4% congestive heart failure, 15.2% stroke/TIA and 14.1% thromboembolism (Table 1 and Fig 2). These proportions were similar across OAC cohorts; however, stroke/TIA appeared to be higher in the apixaban cohort (19.7%) compared to the other OAC cohorts (ranged 14.2% for VKA to 16.8% for dabigatran). The proportion of patients with high baseline bleeding risk (i.e. HAS-BLED ≥ 3) was slightly higher in the apixaban cohort (72.6% apixaban; 69.6% rivaroxaban; 66.3% dabigatran; 67.1% VKA). The proportions with a history of bleeding-related events were similar across cohorts (composite of gastrointestinal (GI) bleed, GI ulceration, intracranial bleed, other bleeds and any bleed). Concomitant therapy with parenteral anticoagulants was identified among 15.5% of VKA patients, while this was less than 4% in each of the NOAC cohorts. A higher proportion of VKA patients (11.9%) were also prescribed concomitant antiplatelet therapy compared to the NOAC cohorts (ranged 7.8% for apixaban to 9.4% for rivaroxaban).

### Persistence

Of 15,244 patients, 12,545 (82.3%) had sufficient follow-up for persistence assessment (i.e. > 60 days between index date and end of follow-up; n = 926 in the apixaban cohort, n = 4,720 rivaroxaban, n = 1,367 dabigatran, n = 5,532 VKA). Median follow-up was longest in the dabigatran cohort (median 12.6 months, IQR 7.3–17.3) and shortest in the apixaban cohort (median 7.1 months, IQR 4.2–11.1). The median prescription durations were 30 days for apixaban, 98 days for rivaroxaban, 50 days for dabigatran and 100 days for VKA.

**Fig 3:**
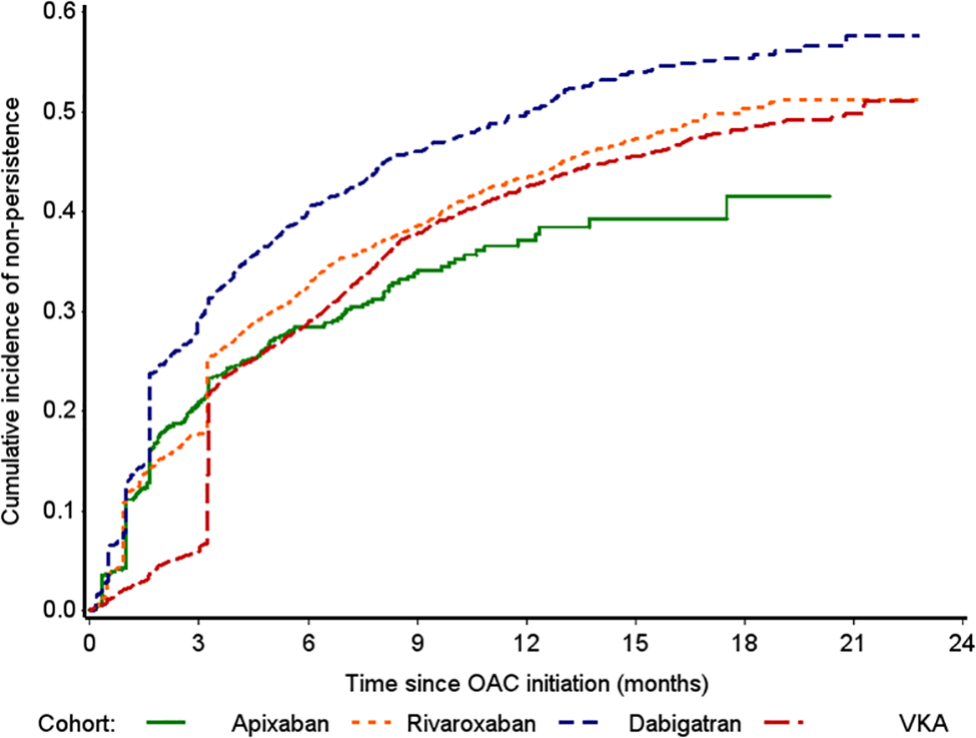
Cumulative incidence of OAC non-persistence.

The persistence rates presented in Table 2, and the cumulative incidence rates of non-persistence in Fig 3, show that the observed patterns of persistence changed over time. In the early months following treatment initiation, it appears that VKA persistence was highest, with 94.0% (95% CI 93.4–94.6%) of patients being persistent. However, Fig 3 shows there was a sharp rise in VKA non-persistence (i.e. a decline in VKA persistence) after the first three months. Overall, the cumulative incidence of non-persistence with dabigatran was the highest of the four cohorts over the entire follow-up period (Fig 3). At 12 months, 62.9% (95% CI 58.8–67.0%) of apixaban patients were persistent, followed by 57.5% of VKA patients (95% CI 56.0–59.0%), 56.6% of rivaroxaban patients (95% CI 54.9–58.2%) and 50.1% of dabigatran patients (95% CI 47.2–53.1%) (Table 2).

**Table 2:** Cumulative incidence of persistence rates at specified time points.

	All NOACs	Apixaban	Rivaroxaban	Dabigatran	VKA
	N = 7,013	N = 926	N = 4,720	N = 1,367	N = 5,532
**Persistence at different time points**					
** At 3 months**					
% (95% CI)^#^	80.7 (80.0–81.4)	79.1 (76.4–81.7)	82.3 (81.2–83.4)	71.1 (68.7–73.5)	94.0 (93.4–94.6)
N at risk	9,091	643	3,606	908	4,896
N censored	829	93	283	68	308
** At 6 months**					
% (95% CI)^#^	67.5 (66.7–68.4)	71.6 (68.4–74.7)	67.3 (65.8–68.7)	59.8 (57.1–62.5)	71.1 (69.8–72.4)
N at risk	5,915	362	2,289	655	2,937
N censored	2,641	323	990	184	1,133
** At 12 months**					
% (95% CI)^#^	57.3 (56.2–58.3)	62.9 (58.8–67.0)	56.6 (54.9–58.2)	50.1 (47.2–53.1)	57.5 (56.0–59.0)
N at risk	2,678	103	997	351	1,377
N censored	5,171	553	1,999	399	2,230
** At end of follow-up**					
% (95% CI)^#^	47.5 (45.6–49.4)	58.5 (52.3–64.9)	48.8 (46.6–51.1)	42.4 (38.7–46.4)	48.9 (46.6–51.3)
N at risk	0	0	0	0	0
N censored	7,599	652	2,909	715	3,492

# 100% minus the cumulative incidence of non-persistence.

These apparent differences in persistence between OAC cohorts were supported by the adjusted comparisons of non-persistence, whereby analyses were controlled for differences in patients’ baseline characteristics across OAC cohorts. Over the entire follow-up period, there was no statistical evidence at the 5% level of a difference in the rate of non-persistence for apixaban compared to VKA (adjusted HR 1.08, 95% CI 0.95–1.24) (Table 3). Non-persistence with rivaroxaban (adjusted HR 1.21, 95% CI 1.14–1.29) and dabigatran (adjusted HR 1.53, 95% CI 1.40–1.68) was more likely compared to VKA. However, the cumulative incidence of non-persistence by OAC shows that patterns changed at 100 days’ follow-up (Fig 3), which was supported by reassessing Schoenfeld residuals in the periods before and after 100 days. After applying the post-hoc time-partitioned approach, VKA non-persistence in the first 100 days was less likely compared to all three NOACs (Table 3); however, after 100 days of treatment, compared to VKA, non-persistence was less likely for apixaban (adjusted HR 0.66, 95% CI 0.52–0.85) and similar for rivaroxaban and dabigatran. In comparison with apixaban, non-persistence was similar with rivaroxaban (adjusted HR 1.03, 95% CI 0.89–1.20) but higher with dabigatran in the first 100 days of treatment (adjusted HR 1.39, 95% CI 1.17–1.66), while after 100 days, non-persistence was more likely with both rivaroxaban (adjusted HR 1.46, 95% CI 1.13–1.88) and dabigatran (adjusted HR 1.67, 95% CI 1.26–2.19).

**Table 3:** Comparison of non-persistence overall and using a time-partitioned approach in final, adjusted model.

	Hazard ratio	95% confidence interval		P-value
		Lower	Upper	
**Overall non-partitioned model (N = 12,545)**				
**Index medication (reference category: VKA)**				
Apixaban	1.08	0.95	1.24	0.237
Rivaroxaban	1.21	1.14	1.29	<0.001
Dabigatran	1.53	1.40	1.68	<0.001
**Demographics**				
Age at index date (years)	0.99	0.99	1.00	<0.001
Region (East vs. West Germany)	0.83	0.77	0.90	<0.001
**History of stroke risk factors (yes vs. no)**				
Stroke or transient ischaemic attack	0.83	0.77	0.91	<0.001
Hypertension	0.78	0.72	0.84	<0.001
Diabetes	0.91	0.86	0.97	0.002
**Concomitant therapy**^**^**^ **(yes vs. no)**				
Parenteral anticoagulants	1.13	1.02	1.24	0.022
Aspirin	1.35	1.23	1.49	<0.001
Other antiplatelet	1.18	1.03	1.35	0.016
**Time-partitioned model**				
***First 100 days follow-up (N = 12*,*545)***				
**Index medication (reference category: VKA)**				
Apixaban	1.37	1.17	1.59	<0.001
Rivaroxaban	1.41	1.30	1.53	<0.001
Dabigatran	1.91	1.70	2.14	<0.001
**Demographics**				
Region (East vs. West Germany)	0.78	0.70	0.86	<0.001
**History of stroke risk factors (yes vs. no)**				
Stroke or transient ischaemic attack	0.82	0.73	0.91	<0.001
Vascular disease	0.90	0.83	0.97	0.005
Hypertension	0.78	0.71	0.85	<0.001
Diabetes	0.91	0.85	0.99	0.024
**Concomitant therapy**^**^**^ **(yes vs. no)**				
Parenteral anticoagulants	1.23	1.08	1.40	0.002
Aspirin	1.40	1.24	1.58	<0.001
Other antiplatelet	1.24	1.04	1.47	0.014
***After the first 100 days of follow-up (N = 8*,*468)***				
**Index medication (reference category: VKA)**				
Apixaban	0.66	0.52	0.85	0.001
Rivaroxaban	0.97	0.87	1.07	0.496
Dabigatran	1.10	0.95	1.28	0.185
**Demographics**				
Age at index date (years)	0.99	0.99	1.00	<0.001
**History of stroke risk factors (yes vs. no)**				
Stroke or transient ischaemic attack	0.86	0.76	0.99	0.030
Hypertension	0.77	0.68	0.87	<0.001
**Concomitant therapy**^**^**^ **(yes vs. no)**				
Aspirin	1.35	1.16	1.58	<0.001

^ Concomitant therapy: prescribed on index date or within 3 months after index date.

### Sensitivity analyses

In the first sensitivity analysis, we identified 10,115 (66.4%) patients who regularly visited the physician (i.e. at least once every 3 months) in the 12 months prior to index date. Limiting the persistence analysis to this sub-group of patients (n = 8,780 with sufficient follow-up for persistence assessment) made little difference to the observed pattern of persistence across the OACs.

**Fig 4:**
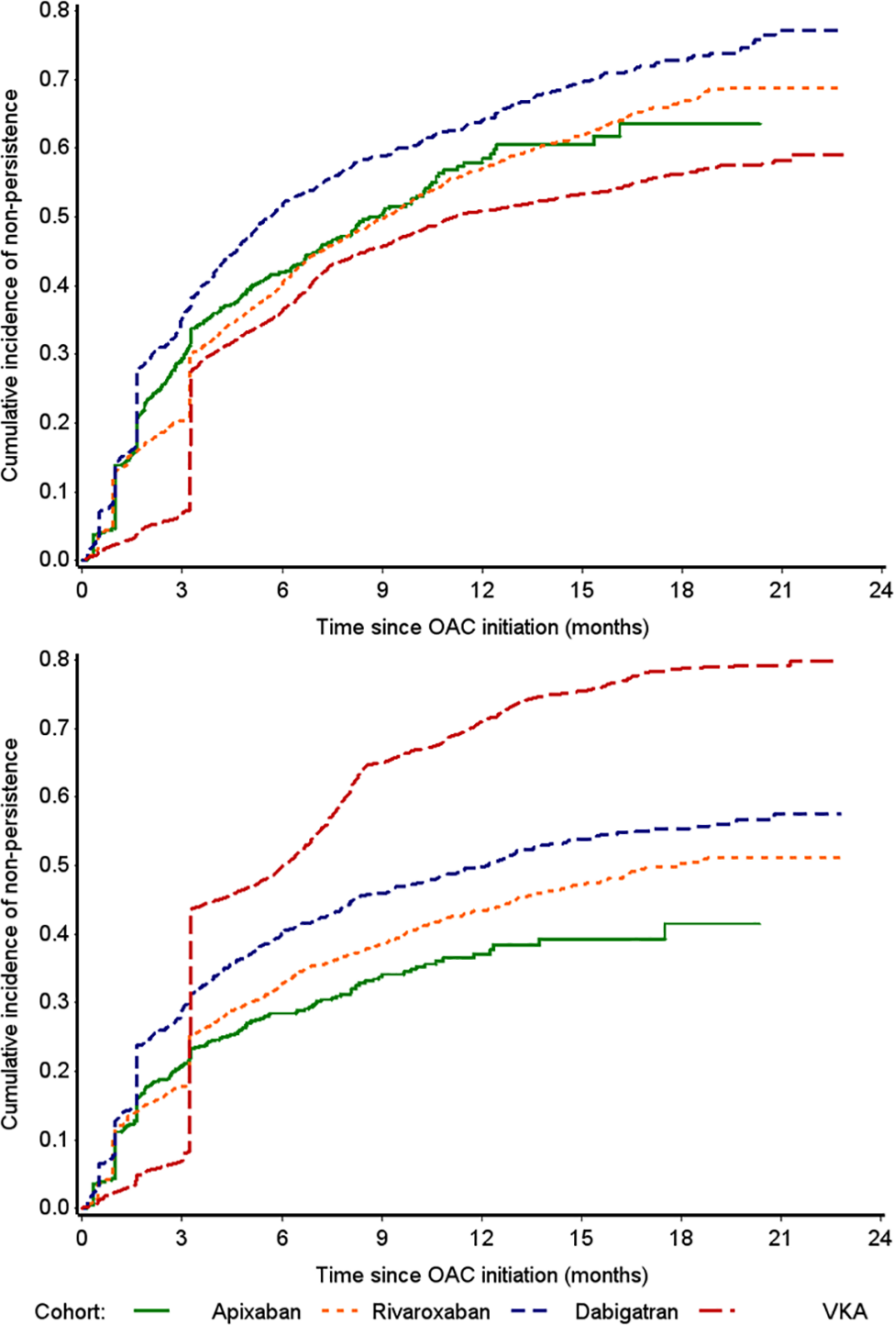
Cumulative incidence of OAC non-persistence in sensitivity analyses (A) using a discontinuation gap of 30 days and (B) when INR records are not used to estimate VKA treatment.

The effect of a change to the definition of the discontinuation gap from 60 days to 30 days is shown in Fig 4 (panel A). As expected, given that shortening the gap allows less time for a subsequent prescription to be identified, there was an increase in non-persistence for all OACs; however, there was a notable change in the pattern of persistence with VKA appearing to show similar or lower rates of non-persistence than all three NOACs across the entire follow-up period.

In the final sensitivity analysis, the removal of INR records to extend VKA treatment increased VKA non-persistence, as shown in Fig 4 (panel B). The patterns of persistence are similar to the main analysis up to 100 days’ follow-up; however, after 100 days, VKA non-persistence increased dramatically and appeared higher than all three NOACs.

## Discussion

### Key findings

This study examined the characteristics and persistence of patients with NVAF newly prescribed stroke preventative OACs between 1st December 2012 and 31st October 2014 in Germany. While patients receiving OACs commonly have a history of stroke risk factors, high stroke and bleeding risk, this study indicates potential differential prescribing whereby apixaban may be prescribed more often than other OACs to patients with a history of stroke/TIA and a high bleeding risk. Persistence patterns appear to change over time, noting that this may be an artefact of the data as discussed below; however, there is evidence that after an initial 100 days of treatment, persistence with apixaban is higher than with rivaroxaban, dabigatran and VKA.

This study used routine clinical data from German primary care. Patients with NVAF are often managed by the primary care physician who is responsible for prescribing stroke preventative treatments and for monitoring patients’ stroke and bleeding risk. Such information is expected to be recorded in primary care data and has therefore enabled the assessment of the real-world use of OACs for stroke prevention in NVAF in Germany.

### Patient characteristics

There are few real-world studies which have examined the characteristics and persistence of patients prescribed the OACs currently available for NVAF. Similar to our earlier study in the UK, we observed a greater proportion of patients with history of stroke/TIA in the apixaban group [21]. While, in the UK study, the apixaban cohort did not have the highest baseline bleeding risk, there did appear to be a higher proportion with history of bleeding compared to the other OACs. In a Swedish study of persistence by Forslund *et al*, a real-world study of claims data in the Stockholm region, they also observed slightly more patients with a history of stroke/TIA in the apixaban group [22]. In Denmark, Olesen *et al* performed a descriptive study of OAC naïve patients between 2011 and 2013 using real-world data gathered from the Danish nationwide administrative registers [23], where the history of stroke/TIA was similar across OAC cohorts; however, previous bleeding events were most frequent among apixaban patients. In the US, two large claims database studies had different findings from our study, showing that patients with greater comorbidities were prescribed VKA over apixaban, rivaroxaban and dabigatran [24,25]. In another US claims data study of the NOACs, there were more apixaban patients with a history of stroke compared to rivaroxaban and dabigatran, however the overall proportion with a previous stroke was much lower than observed in Germany [26]. In contrast to our findings, a low proportion of patients had a high risk of bleeding at baseline (less than 15% in each NOAC cohort compared to, overall, 68.4% in our study). This may be due to a difference in methods as we calculated HAS-BLED scores based on information from all medical history available in the data, to ensure we captured long-term conditions, while the US study based the score on information from the previous 12 months. In Germany, recent conference proceedings of a study examining the safety of OACs in NVAF found slightly younger patients prescribed rivaroxaban and dabigatran compared to our study [27]. Patients in their apixaban and VKA cohorts had a higher average CHA_2_DS_2_-VASc score than rivaroxaban and dabigatran, which is in line with our results where there was a larger proportion of patients with a high stroke risk among patients prescribed apixaban and VKA.

A recent study in Germany by Beyer-Westendorf *et al* examined persistence with rivaroxaban, dabigatran and VKA in patients with NVAF using the same data as used in our study, IMS® Disease Analyzer [28]. Average age was similar to patients in our study but the gender distribution differed slightly. The mean CHA_2_DS_2_-VASc scores were similar to our study. While the setting of this and our study are similar, there were several methodological improvements in our study design which we believe enhance the credibility of our findings. These include: 1) our study was over a longer period, thus allowing us to include apixaban in addition to rivaroxaban and dabigatran, 2) we used a longer minimum length of data availability in order to capture more complete information on patients’ medical history, and 3) we conservatively allowed for possible additional prescribing of VKA outside of primary by assuming they were prescribed VKA when a INR test was performed and not only when a prescription was recorded.

In the 2012 updated clinical guideline from the European Society of Cardiology, NOACs were broadly recommended over VKAs for stroke prevention in NVAF [29]. Potential differences in patient characteristics across OAC cohorts in our study suggest that there may be also be favoured prescribing of apixaban over other NOACs to patients with a history of stroke/TIA and higher bleeding risk. It may also be that particular preferences have been influenced by the ARISTOTLE clinical trial which indicated better stroke prevention and fewer bleeds in with apixaban compared with warfarin [6,16]. The exact reasons why physicians may be prescribing apixaban more than the other OACs to specific groups of patients cannot be derived from the data. These potential differences in patient profiles need to be confirmed in larger studies and are important to consider when conducting drug-specific outcomes research since they may lead to channelling bias.

### Persistence

After 12 months’ treatment, the rate of persistence with apixaban was slightly higher than the other OACs. While inferences from the 12 month persistence rates are limited due to short follow-up, particularly in the apixaban cohort (103 patients assessed at 12 months compared to 997 rivaroxaban, 351 dabigatran and 1,377 VKA patients), it does give us some indication of the relative differences in persistence which can be useful in comparing against other research findings. Studies in the UK, Sweden and the US found 12 month persistence rates for the NOACs higher than those observed in this study [11,22,30], while in the German study by Beyer-Westendorf *et al*, the rates for rivaroxaban and dabigatran were similar to ours (apixaban was not included in their study) [28]. VKA persistence rates were lower in two studies [11,28], probably because we extended VKA treatment continuity in our study if an INR test was recorded; their results are similar to those in our sensitivity analysis without the use of INR records. Contrary to our results using both overall and time-partitioned approaches, several studies have found improved persistence with rivaroxaban and dabigatran over VKA [11,28,31–33] which, again, is likely to be a consequence of using a different method for estimating time under VKA treatment that increases exposure time. This is plausible since the sensitivity analysis of persistence without the use of INR to extend treatment time showed much higher VKA non-persistence, greater than all three NOACs. However, it could also reflect differences between countries in their health care systems, prescribing practices and data sources. Only three studies have compared apixaban persistence with other OACs. Forslund *et al* found persistence between VKA and apixaban to be comparable in Sweden, and higher persistence with apixaban compared to dabigatran and rivaroxaban [22]. Conference proceedings from Pan *et al* suggest improved persistence with apixaban over VKA, rivaroxaban and dabigatran in the US [34]. Similar patterns were also observed in our UK study [21].

The difference in the results of our comparisons to VKA and those from previously published studies brings to light two methodological aspects of our study which do not appear to be considered in many other studies. Firstly, as previously mentioned, the assumption that a VKA prescription is provided outside of primary care (and therefore not included in the data) if there is a record of an INR test. These tests are performed routinely, outside of the primary care setting, in patients receiving VKA treatment and contribute towards determining whether there should be dose alterations or additional prescriptions of VKA. Without accounting for this possibility, treatment duration could be underestimated. Conversely, our cautious approach may overestimate VKA duration, which may explain why, unlike other studies, we did not observe higher persistence with rivaroxaban and dabigatran over VKA until we performed the sensitivity analysis using the same approach as other studies, without the use of INR records. Secondly, we cautiously addressed the non-proportionality of the hazards by performing a time-partitioned analysis whereby follow-up was separated into two periods before and after 100 days. Using the non-partitioned model, it appeared that apixaban persistence was similar to VKA. However, we see that this is not the case but the result of averaging the effect over the entire follow-up period. The time-partitioned approach revealed increased persistence for VKA over apixaban in the first 100 days, but in long-term treatment after the initial 100 days, persistence with apixaban superseded that of VKA. It is not clear if this approach was considered in other studies as it has not been reported in previous publications.

It is worth noting that the results comparing time to non-persistence in the first 100 days following treatment, which suggest VKA persistence was greater than all other OACs, was potentially related to the working definition of discontinuation. In our data, over 90% of the VKA prescriptions were for 98 or 100 pills, thus lasting approximately 100 days assuming that one pill a day was prescribed. Therefore, in the initial 100 days of follow-up, we only observe VKA non-persistence if there was a switch to another OAC. At 100 days, we see a sharp rise in VKA non-persistence because it includes all discontinuation of VKA which occurred at any point during the first 100 days.

There are other reasons which may explain differences in persistence results between studies, some particular to OAC treatment (e.g. local VKA treatment infrastructure). Different countries have different health care systems; in the UK, anticoagulation clinics exist to monitor patients on VKA, which may encourage patients to continue medication and lead to higher persistence rates. The databases used in real-world studies also vary in the data collected. In the present study, only prescriptions issued in primary care were available; prescriptions issued elsewhere were not identifiable. This differs to claims databases which have been used in Sweden and the US, where there may be more complete recording of prescribing from the use of pharmacy dispensed medication data [22,32]. Data on prescription daily doses and duration also vary between databases and studies. In this study, we had to assume daily doses in order to calculate prescription duration and therefore total length of time on treatment. Other databases may hold more information or made different assumptions on prescription durations. For example, in the Beyer-Westendorf study, it was assumed that patients on VKA were prescribed a daily dose of 3mg/day [28] while we assumed one pill a day was prescribed. Both assumptions are valid but will lead to slight variations in prescription length. Methods of analysing persistence also differ between studies. In the Swedish study, their method differed because of the reimbursement structure of claims in Sweden [22]; persistence was calculated as the proportion of patients alive and treated in six month blocks of follow-up therefore time-to-event analysis was not used. In database studies of treatment persistence, it is necessary to make assumptions and, while this can impede comparisons of results between studies, the results will still be internally valid if they do not bias the analysis in favour of a particular treatment or comparator. However, as mentioned previously, our study may have favoured VKA in the main analyses by extending the VKA treatment lines; this was to ensure that VKA use outside of primary care was not underestimated.

It is interesting to observe that apixaban persistence is higher than other OACs (albeit after the first 100 days since initiation) as it has been previously speculated that the once-a-day regimen is more convenient than twice-a-day regimens and thus probably lead to better persistence [35]. Our study shows that persistence is likely affected by other factors as well. One possible explanation for observing improved persistence with apixaban which has a twice-a-day regimen is that these patients may have greater severity of AF and/or comorbidities (as observed in our study) which could increase patients’ understanding of the importance of treatment continuation.

We cannot derive clear reasons which explain differences in non-persistence behaviour between OACs using this database alone. It is possible that in the early period of VKA treatment, persistence with VKA may be better than with the NOACs because patients prescribed VKA have to be regularly seen and monitored. However, as mentioned earlier, high VKA persistence in the early period is likely to be driven by the data. While apixaban and VKA have differing modes of action to prevent stroke, this study cannot directly attribute differences in non-persistence with the pathophysiological mechanisms of the drugs. Rather, it may be that non-persistence is lower with apixaban than VKA (as observed in the period after 100 days treatment) due to patient and physician preferences; the ARISTOTLE trial indicated reduced risk of stroke and fewer bleeds with apixaban compared to VKA patients [6,16], which may give apixaban a more favourable profile that encourages greater persistence. The findings from the ARISTOTLE trial have been further supported by a recent large observational study in the US, however the later timing of their results would not have influenced prescribing observed in our study [24].

This study did not investigate safety or effectiveness of OACs, nor reasons for non-persistence; rather, it highlighted that a large proportion of patients stop or switch OACs. An area needing additional research is why non-persistence occurs and whether it is appropriate. For example, non-persistence would be expected if a patient undergoes cardioversion where cessation of OACs is a requirement. However, reasons for non-persistence could also highlight problems with tolerability and effectiveness of a drug. Common reasons for discontinuation can include the occurrence of a major bleed or worries about bleeding, and physician and patient preferences [36].

There are limitations to this study which should be acknowledged. The nature of the health care system in Germany means that people can see a specialist without referral from a primary care physician. As such, those who are treated solely by a specialist are not captured in the data, and importantly, prescriptions issued by a specialist will not be recorded. Furthermore, consultations with a different primary care physician are not recorded. This would mean that non-persistence was overestimated in the study and would also have led to misclassification of patients as OAC-naïve if they had previously been prescribed elsewhere. Unmeasured data are an inherent issue with routine clinical data and are a particular problem if potential confounders are not captured. In this data, missing information on death and transfer to another physician may have impacted the amount of follow-up and therefore time to non-persistence. Daily dose was largely missing in this data and the standard daily dose was otherwise assumed. Accurate information on all aspects of an OAC prescription are needed to provide greater precision to the persistence estimates. Missing data (e.g. creatinine clearance, BMI) and lack of detail on diagnoses are also limitations of the data. This study does, however, also have a number of strengths. It is a real-world study and while there are limitations with such data as described above, the use of real-world data enables us to observe how OACs are being prescribed and the continuity of their use in routine practice outside of the clinical trial setting. This is of particular importance as new drugs, such as apixaban, are introduced into routine care; this study is the first to describe apixaban use for NVAF in Germany. As stated earlier, we have enhanced the credibility of our findings with the study design; for example, we increased VKA continuation where INR tests were recorded to account for changes to prescribing unobserved in primary care data, and used a time-partitioned approach when we examined and found evidence of non-proportionality.

## Conclusions

This study found evidence of higher persistence with apixaban than with rivaroxaban, dabigatran, or VKA after the first 100 days of treatment in German primary care while treatment during the first 100 days was more persistent with VKA than other OACs. This study also found potential differences in characteristics of patients with NVAF prescribed different OACs, which may impact channelling bias in research into OACs. Overall, this area of research would benefit from additional studies with larger cohorts with longer follow-up. Future studies could also examine reasons for non-persistence, the relationship between persistence and dosage, and adverse outcomes such as bleeding events associated with OAC use.

## Supporting information

S1 Table Patient characteristics for OAC-naïve and OAC-experienced patients. (DOCX)

S2 Table Cumulative incidence of persistence rates at specified time points among OAC-naïve and OAC-experienced patients. (DOCX)
