# Supplementary material for: Oral anticoagulant persistence in patients with non-valvular atrial fibrillation: A cohort study using primary care data in Germany
Source: PLoS One. 2017 Oct 10;12(10):e0185642. doi: 10.1371/journal.pone.0185642 (PMC5634552; doi:10.1371/journal.pone.0185642)
Supplement: S1 Table — (DOCX) [file pone.0185642.s001.docx]

**S1 Table. Patient characteristics for OAC-naïve and OAC-experienced patients.**

|  | **All study population** | **Apixaban** | **Rivaroxaban** | **Dabigatran** | **VKA** |
| --- | --- | --- | --- | --- | --- |
|  | **N=20,427** | **N=2,678** | **N=9,562** | **N=2,696** | **N=7,215** |
| **Gender (n, %)** |  |  |  |  |  |
| Male | 10,479 (51.3%) | 1,315 (49.1%) | 4,778 (50.0%) | 1,352 (50.1%) | 3,812 (52.8%) |
| Female | 9,948 (48.7%) | 1,363 (50.9%) | 4,784 (50.0%) | 1,344 (49.9%) | 3,403 (47.2%) |
| **Region (n, %)** |  |  |  |  |  |
| West Germany | 16,535 (80.9%) | 2,194 (81.9%) | 7,563 (79.1%) | 2,177 (80.7%) | 6,038 (83.7%) |
| East Germany | 3,892 (19.1%) | 484 (18.1%) | 1,999 (20.9%) | 519 (19.3%) | 1,177 (16.3%) |
| **Age (years) at index date (n, %)** | N*=22,151 |  |  |  |  |
| ≥ 70 | 16,615 (75.0%) | 2,100 (78.4%) | 7,127 (74.5%) | 2,012 (74.6%) | 5,376 (74.5%) |
| Median (IQR) | 76 (70-82) | 77 (71-83) | 76 (69-82) | 76 (69-82) | 75 (69-81) |
| **Time (months) between AF diagnosis and index date** | N*=22,151 |  |  |  |  |
| Median (IQR) | 4.2 (0.0-35.0) | 15.4 (0.2-47.0) | 10.1 (0.1-43.3) | 7.7 (0.1-40.2) | 1.0 (0.0-15.2) |
| **History of stroke risk factors (n, %)** | N*=22,151 |  |  |  |  |
| Stroke or transient ischaemic attack | 3,855 (17.4%) | 626 (23.4%) | 1,660 (17.4%) | 527 (19.5%) | 1,042 (14.4%) |
| Thromboembolism | 3,867 (17.5%) | 490 (18.3%) | 1,876 (19.6%) | 431 (16.0%) | 1,070 (14.8%) |
| Congestive heart failure | 8,767 (39.6%) | 1,164 (43.5%) | 3,913 (40.9%) | 1,067 (39.6%) | 2,623 (36.4%) |
| Vascular disease | 12,929 (58.4%) | 1,641 (61.3%) | 5,598 (58.5%) | 1,543 (57.2%) | 4,147 (57.5%) |
| Hypertension | 18,979 (85.7%) | 2,361 (88.2%) | 8,168 (85.4%) | 2,329 (86.4%) | 6,121 (84.8%) |
| Diabetes | 8,800 (39.7%) | 1,111 (41.5%) | 3,818 (39.9%) | 1,061 (39.4%) | 2,810 (38.9%) |
| **CHA_2_DS_2_-VASc score at index date (n, %)** | N*=22,151 |  |  |  |  |
| < 2 | 1,101 (5.0%) | 107 (4.0%) | 518 (5.4%) | 150 (5.6%) | 326 (4.5%) |
| ≥ 2 | 21,050 (95.0%) | 2,571 (96.0%) | 9,044 (94.6%) | 2,546 (94.4%) | 6,889 (95.5%) |
| **History of events (n, %)** | N*=22,151 |  |  |  |  |
| Gastrointestinal ulceration | 1,524 (6.9%) | 198 (7.4%) | 672 (7.0%) | 183 (6.8%) | 471 (6.5%) |
| Gastrointestinal bleeding | 3,813 (17.2%) | 514 (19.2%) | 1,710 (17.9%) | 499 (18.5%) | 1,090 (15.1%) |
| Other bleeding^¶^ | 2,305 (10.4%) | 354 (13.2%) | 1,096 (11.5%) | 277 (10.3%) | 578 (8.0%) |
| Any bleeding^¶^ | 5,801 (26.2%) | 804 (30.0%) | 2,653 (27.7%) | 731 (27.1%) | 1,613 (22.4%) |
| **HAS-BLED score^#^ at index date (n, %)** | N*=22,151 |  |  |  |  |
| < 3 | 6,336 (28.6%) | 553 (20.6%) | 2,550 (26.7%) | 799 (29.6%) | 2,434 (33.7%) |
| ≥ 3 | 15,815 (71.4%) | 2125 (79.4%) | 7,012 (73.3%) | 1,897 (70.4%) | 4,781 (66.3%) |
| **Concomitant therapy^^^ (n, %)** | N*=22,151 |  |  |  |  |
| Parenteral anticoagulants | 1,632 (7.4%) | 78 (2.9%) | 334 (3.5%) | 107 (4.0%) | 1,113 (15.4%) |
| Antiplatelet | 2,114 (9.5%) | 186 (6.9%) | 804 (8.4%) | 243 (9.0%) | 881 (12.2%) |
| Aspirin monotherapy | 1,674 (7.6%) | 145 (5.4%) | 689 (7.2%) | 190 (7.0%) | 650 (9.0%) |
| Other antiplatelet therapies^ⱡ^ | 843 (3.8%) | 69 (2.6%) | 250 (2.6%) | 91 (3.4%) | 433 (6.0%) |
| Anti-arrhythmic | 2,761 (12.5%) | 333 (12.4%) | 1,236 (12.9%) | 339 (12.6%) | 853 (11.8%) |
| Beta-blocker | 13,676 (61.7%) | 1,560 (58.3%) | 5,838 (61.1%) | 1,634 (60.6%) | 4,644 (64.4%) |
| Statin | 5,875 (26.5%) | 762 (28.5%) | 2,378 (24.9%) | 726 (26.9%) | 2,009 (27.8%) |
| Antidiabetic agent | 3,577 (16.1%) | 422 (15.8%) | 1,505 (15.7%) | 429 (15.9%) | 1,221 (16.9%) |
| Antihypertensive agent | 15,015 (67.8%) | 1,837 (68.6%) | 6,310 (66.0%) | 1,832 (68.0%) | 5,036 (69.8%) |
| Proton pump inhibitor | 6,964 (31.4%) | 844 (31.5%) | 3,023 (31.6%) | 904 (33.5%) | 2,193 (30.4%) |

'N*' represents the number of OAC exposures as patients can have multiple OAC exposures during the study period and be in multiple cohorts. However, gender and region do not vary across exposures and are therefore reported once for each patient (i.e. 'N' represents the number of patients).

¶ Other bleeding includes intraocular, pericardial, urinary, intra-articular and lung bleedings. Any bleeding includes gastrointestinal, intracranial and other bleeding.

# Labile international normalised ratio is also a component of the HAS-BLED score but was not included as there is incomplete recording in IMS® Disease Analyzer. The HAS-BLED score therefore ranges from 0 to 8. High alcohol intake has been included in the HAS-BLED score however is likely under-recorded in IMS® Disease Analyzer.

^ Concomitant therapy: prescribed on index date or within 3 months after index date.

ⱡ Other antiplatelet therapy includes abciximab, clopidogrel, dipyridamole, prasugrel, ticagrelor, ticlopidine and tirofiban.
