# Supplementary material for: Oral anticoagulant persistence in patients with non-valvular atrial fibrillation: A cohort study using primary care data in Germany
Source: PLoS One. 2017 Oct 10;12(10):e0185642. doi: 10.1371/journal.pone.0185642 (PMC5634552; doi:10.1371/journal.pone.0185642)
Supplement: S2 Table — (DOCX) [file pone.0185642.s002.docx]

**S2 Table. Cumulative incidence of persistence rates at specified time points among OAC-naïve and OAC-experienced patients.**

|  | **All NOACs** | **Apixaban** | **Rivaroxaban** | **Dabigatran** | **VKA** |
| --- | --- | --- | --- | --- | --- |
|  | **N=12,268** | **N=1,997** | **N=7,962** | **N=2,309** | **N=6,060** |
| **Persistence at different time points** |  |  |  |  |  |
| **At 3 months** |  |  |  |  |  |
| % (95% CI)^#^ | 80.7 (80.0-81.4) | 81.6 (79.8-83.3) | 83.3 (82.5-84.1) | 70.9 (69.0-72.7) | 93.8 (93.1-94.4) |
| N at risk | 9,091 | 1,433 | 6,139 | 1,519 | 5,343 |
| N censored | 829 | 202 | 503 | 124 | 342 |
| **At 6 months** |  |  |  |  |  |
| % (95% CI)^#^ | 67.5 (66.7-68.4) | 71.9 (69.7-74.0) | 69.1 (68.0-70.1) | 59.0 (57.0-61.1) | 71.0 (69.7-72.2) |
| N at risk | 5,915 | 811 | 4,006 | 1,098 | 3,182 |
| N censored | 2641 | 680 | 1,655 | 306 | 1,271 |
| **At 12 months** |  |  |  |  |  |
| % (95% CI)^#^ | 57.3 (56.2-58.3) | 63.4 (60.5-66.2) | 58.3 (57.0-59.6) | 49.6 (47.4-51.9) | 57.5 (56.0-58.9) |
| N at risk | 2,678 | 229 | 1,844 | 605 | 1,469 |
| N censored | 5171 | 1,202 | 3,318 | 651 | 2,490 |
| **At end of follow-up** |  |  |  |  |  |
| % (95% CI)^#^ | 47.5 (45.6-49.4) | 54.6 (47.0-62.6) | 49.6 (47.8-51.5) | 38.4 (34.4-42.8) | 48.8 (46.6-51.1) |
| N at risk | 0 | 0 | 0 | 0 | 0 |
| N censored | 7,599 | 1,419 | 4,998 | 1,182 | 3,833 |

# 100% minus the cumulative incidence of non-persistence.
